# Physical Sensing of Surface Properties by Microswimmers – Directing Bacterial Motion via Wall Slip

**DOI:** 10.1038/srep09586

**Published:** 2015-05-20

**Authors:** Jinglei Hu, Adam Wysocki, Roland G. Winkler, Gerhard Gompper

**Affiliations:** 1 Theoretical Soft Matter and Biophysics, Institute of Complex Systems and Institute for Advanced Simulation, Forschungszentrum Jülich, D-52425 Jülich, Germany

## Abstract

Bacteria such as *Escherichia coli* swim along circular trajectories adjacent to surfaces. Thereby, the orientation (clockwise, counterclockwise) and the curvature depend on the surface properties. We employ mesoscale hydrodynamic simulations of a mechano-elastic model of *E. coli*, with a spherocylindrical body propelled by a bundle of rotating helical flagella, to study quantitatively the curvature of the appearing circular trajectories. We demonstrate that the cell is sensitive to nanoscale changes in the surface slip length. The results are employed to propose a novel approach to directing bacterial motion on striped surfaces with different slip lengths, which implies a transformation of the circular motion into a snaking motion along the stripe boundaries. The feasibility of this approach is demonstrated by a simulation of active Brownian rods, which also reveals a dependence of directional motion on the stripe width.

Motility is a particular property of flagellated prokaryotic microorganisms, which are ubiquitous in nature. Most bacteria exploit helical filaments for propulsion, driven by rotary motors located in their cell membrane. Prominent examples of peritrichous bacteria, which possess numerous flagella, are *Escherichia coli, Salmonella typhimurium, Rhizobium lupini*, or *Proteus mirabilis* to name just a few. In a bulk fluid, the bacteria move in a straight manner (run), with all flagella forming a bundle, interrupted by abrupt changes of the swimming direction (tumble) induced by disintegration of the bundle.^1^ The presence of a surface drastically alters the swimming behavior. For instance, the non-tumbling mutant of *E. coli* swims in a clockwise (CW) circular trajectory close to a solid boundary^2^ and a counterclockwise (CCW) trajectory close to a liquid-air interface.^3^ Hence, bacteria are able to “sense” the properties of a nearby surface, an aspect of paramount importance for surface selection and attachment in the early stages of biofilm formation or infection.^2^,^4^,^5^

The swimming behavior of bacteria near surfaces is governed by hydrodynamic forces^6^,^7^ and, hence, the CW and CCW circular trajectories of *E. coli* have been explained in terms of hydrodynamic interactions.^2^,^3^ Physically, the resulting trajectory is controlled by the fluid slip on the surface—CW trajectories follow for no-slip and CCW for perfect-slip boundary conditions. Usually, a surface exhibits partial slip due to adsorbents, microstructures, and hydrophobicity.^8^,^9^ A quantified measure of slip is the slip length *b* as the extrapolated distance below the surface, where the fluid velocity vanishes.^10^ By definition, *b* = 0 for no-slip and *b* = ∞ for perfect-slip surfaces. Intuitively, a trajectory can be expected to switch from CW to CCW (or vice versa) when *b* reaches some characteristic value *b*_0_. Such a transition has been observed experimentally for *E. coli* swimming near glass surfaces upon addition of alginate, and has been attributed to changes in the slip length.^11^ An attempt of a unified description has been presented, based on the far-field approximation of hydrodynamic interactions.^12^ Yet, there is no quantitative theoretical or simulation study on the effect of slip on the swimming behavior of bacteria at surfaces. A theoretical understanding of hydrodynamic interactions between swimming bacteria and surfaces not only sheds light on selective surface attachment, but opens an avenue for the design of microfluidic devices to control and guide bacterial motion^13^ for separation, trapping, stirring, etc.

Here, we exploit a mesoscale model of a non-tumbling *E. coli*-type bacterium to study its motion near surfaces, and present results for its circular dynamics at surfaces of various slip lengths, see Fig. 1. The simulation fully accounts for hydrodynamic interactions on length scales much smaller than the diameter of the cell, i.e., near-field hydrodynamics, which is very important for a quantitative theoretical description of the phenomenon. We find that *E. coli* senses the nanoscale slip length of the surface and responds with a circular trajectory of a particular radius. The obtained dependence of the curvature on the slip length is well described by a simple derived theoretical expression. Moreover, we employ these insights to suggest a novel route to direct bacterial motion by patterning a surface with stripes of different slip lengths and corresponding CW and CCW trajectories, respectively. This leads to an preferential snaking motion along the stripe boundaries for sufficiently wide stripes. We demonstrate the viability of this approach by a simulation of active Brownian rods, and elucidate the dependence of the diffusion anisotropy on the stripe width.

## Results

### Bacterial swimming near homogeneous surfaces

**Figure 1:**
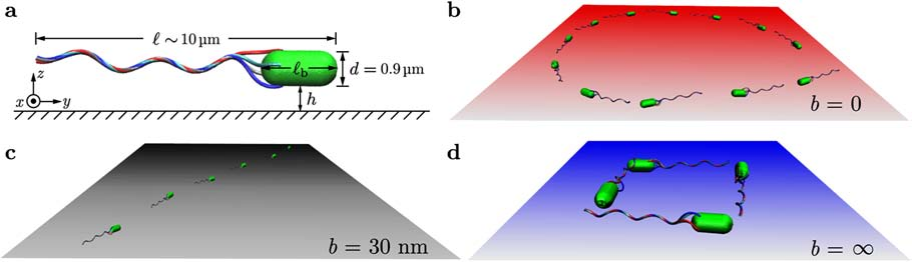
Swimming bacteria sense the slip of its nearby surface. (a) The model bacterium of length 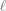 consists of a spherocylindrical body of length 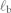 and diameter *d* and four helical flagella each turned by a motor torque. The bacterial geometry and flagellar properties are in agreement with experiments of *E. coli* (Methods and *SI*). The body the flagellar bundle counter rotate. *h* is the gap width between the body and the surface. (b) CW, (c) noisy straight, and (d) CCW trajectories from hydrodynamic simulations of a bacterium swimming near homogeneous surfaces with different slip lengths *b* as indicated.

In our mesoscale hydrodynamic simulations, we model the bacterium *E. coli* by a spherocylindrical body with four attached helical flagella, see Fig. 1(a). The bacterium is immersed in a mesoscale fluid, described by the multiparticle collision dynamics (MPC) technique,^14^,^15^ which itself is confined between two parallel surfaces with tunable slip length. The MPC method includes thermal fluctuations and fully accounts for hydrodynamic interactions down to 100 nm, a length scale much less than the cell-body diameter *d* = 0.9 µm. Rotation of the flagella by applied torques leads to bundle formation^16^,^17^ and swimming motion^18^ (Movie S1 in *SI*). Further details are provided in the Methods section.

**Figure 2:**
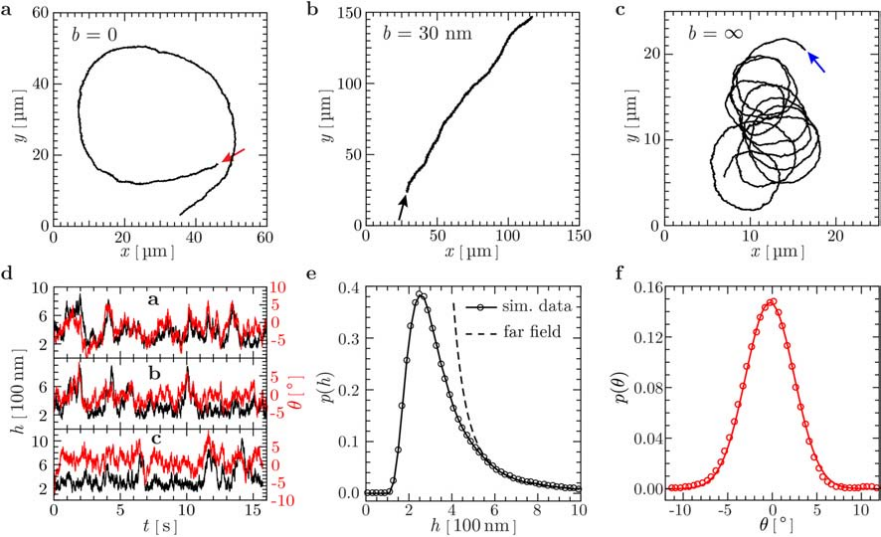
(a)–(c) Simulated trajectories for the *E. coli* model with body length 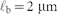, viewed from above the surface with slip length *b* and starting point indicated by the arrow. (d) Time series of the gap width *h* and the inclination angle *θ* between the bacterial swimming direction (pointing from the flagellar to the body center of mass) and the surface. *θ* < 0 if the bacterium swims toward the surface. (e) Comparison of the gap-width distribution obtained from our simulations to the far-field prediction^19^ 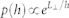 with fit parameter 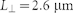. (f) Distribution of the inclination angle *θ*, fitted by a Gaussian distribution with mean 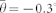 and standard deviation *σ_θ_* = 2.7°.

Near surfaces, bacteria generically swim on circular trajectories due to the counter-rotation of the body and the flagellar bundle. Figures 2(a)–(c) illustrate the simulation trajectories of the *E. coli* model for different slip lengths *b*. The trajectory changes from a CW circle (Fig. 1(b) and Movie S2 in *SI*) to a noisy straight line (Fig. 1(c)) and a CCW circle (Fig. 1(d)) as *b* increases. Figure 2(d) shows that the distance *h* of the body to the surface fluctuates around the average 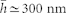 with standard deviation 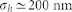. The probability distribution *p*(*h*) is presented in Fig. 2(e). It clearly deviates from the far-field prediction^19^ 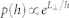, which strongly overestimates the probability for the bacterium to be very close to the surface. The simulation data for *h* > 500 nm are well described by the far-field expression, which provides the length scale 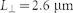.

Our results demonstrate that the far-field approximation fails to quantitatively describe the swimming behavior of bacteria near surfaces. The average distance 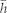 results from the balance of hydrodynamic attraction^19^ and short-range repulsion (which mimics the effect of additional surface-bacterium interactions, see Methods section), independent of the initial position or orientation of *E. coli*. Thus, the resulting height distribution is determined by near-field hydrodynamics, rather than far-field effects. This conclusion is supported by a study of bacterial motion near slip surfaces,^3^ which yields a similar bacterium-surface distance 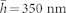 from comparison of experimental data with theoretical results based on mobility tensors of cylindrical and helical bodies. We note that the bacterium has the chance to escape from the surface due to orientational fluctuations, because we see some rare events where it moves from one wall to the other. These events are observed more frequently for larger body lengths, which may be related to the additional active noise due to an increased wobbling motion of the body (compare Movie S1 in *SI*) with increasing 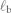. The angle *θ* between the bacterial swimming direction and the surface in Fig. 2(d) deviates by at most 10° from the average, and is found to follow a Gaussian distribution with mean 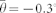 as shown in Fig. 2(f), indicating that the bacterium swims nearly parallel to the surface. These results reflect the importance of noise on the swimming motion of bacteria near surfaces.

**Figure 3:**
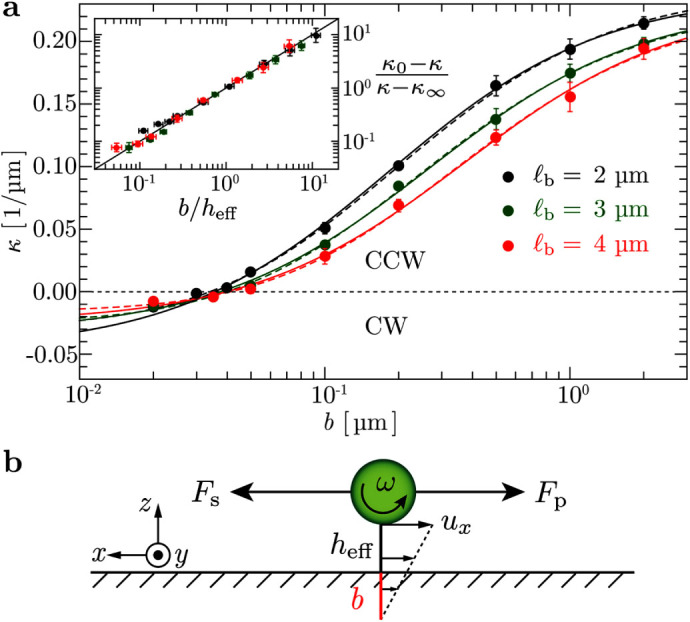
(a) Average curvature *κ* of *E. coli* swimming trajectories vs. surface slip length *b*.The solid and dashed lines are least-square fits of simulation data points to Eq. (1) and the far-field prediction in Ref. 12, respectively. Inset: (*κ*_0_ − *κ*)/(*κ* − *κ*_∞_) vs. *b*/*h*_eff_, with the effective gap width *h*_eff_ obtained from the solid fitted lines in the main plot. The solid line in the inset corresponds to (*κ*_0_ − *κ*)/(*κ* − *κ*_∞_) = *b*/*h*_eff_. (b) Schematic of the model used for analytical calculations for circular swimming trajectories. For a bacterium swimming along the *y*-axis as shown in Fig. 1(a), the cell body rotating around the *y*-axis experiences two forces along the *x*-axis: (i) the shear force *F*_s_ due to the fluid velocity gradient *∂_z_u_x_* ≈ (−*ωd*/2)/(*h*_eff_ + *b*), and (ii) the force *F*_p_ due to pressure difference between the converging (left) and diverging (right) flows in the gap. The bacterium runs CW with *κ* < 0 when *F* = *F*_s_ + *F*_p_ > 0, and CCW with *κ* > 0 when *F* < 0.

The quantitative dependence of swimming trajectories on the slip length is displayed in Fig. 3(a) for different cell-body lengths 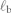. The curvature *κ* for each slip length is an average over up to 10 independent trajectories from extensive hydrodynamic simulations. For each trajectory, the average curvature is the inverse of the circle radius obtained from a least-square fit. The curvature exhibits a smooth crossover from CW trajectories (*κ* < 0) for no-slip surface to CCW trajectories (*κ* > 0). The data are very well described by the analytical expression

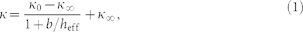
which is based on the consideration of the hydrodynamic lubrication forces on the cell body, which consist of the competing shear force and the force due to the pressure imbalance, as illustrated in Fig. 3(b); see Methods section for a derivation. Here, *κ*_0_ < 0 is the curvature for a no-slip surface (*b* = 0), *κ*_∞_ > 0 for a perfect-slip surface (*b* = ∞), and *h*_eff_ the effective gap width between the cell and the surface. The values of the fit parameters *κ*_0_, *κ*_∞_, and *h*_eff_ are listed in Table 1. The fitted height *h*_eff_ is comparable to the mean gap width 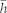, whereas the latter values are somewhat larger, which is attributed to the wobbling motion of the body. The radii of curvature |*κ*_0_|^−1^ and |*κ*_∞_|^−1^ obtained from the fits agree with the values directly measured from our simulations as well as the experimental results.^2^,^11^ As shown in the inset of Fig. 3(a), with the fitted values of *h*_eff_ at different 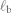, all data points measured from different systems collapse onto a single line (*κ*_0_ − *κ*)/(*κ* − *κ*_∞_) = *b*/*h*_eff_, which is equivalent to Eq. (1). Thus, the results from hydrodynamics simulations are clearly consistent with our theoretical description and experimental results. We like to stress that Eq. (1) is a general result and applies also to other bacteria which exploit rotating flagella for swimming, such as *Bacillus subtilis*, *Salmonella typhimurium* and *Rhodobacter sphaeroides*.

The dependence of the swimming-trajectory curvature on the slip length has been analysed very recently within the far-field approximation for a rotlet dipole, a simplified model for flagellated bacteria with counter-rotation of cell body and flagella.^12^ The trajectory curvature *κ* is found to be a complex function of the dipole strength *q*, the fluid gap width *h*, and the bacterial aspect ratio *γ*; see Eq. (71) in Ref. 12. As displayed in Fig. 3(a), our simulation data can be fitted very well by the far-field prediction, shown by the dashed lines with the fit parameters (*q*/[pN µm^2^], *h*/[nm], *γ*) = (0.002, 259, 2.86), (0.005, 322, 3.00), and (0.016, 441, 3.79) for cell-body length 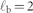, 3, and 4 µm, respectively. The values of *h* are consistent with the mean gap widths in Table 1, whereas the values of *γ* are about three times less than the bacterial aspect ratio 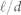, but close to the body aspect ratio 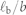. From dimensional analysis, a reasonable estimate of *q* is *q* ≈ 1.5 pN µm^2^, obtained from the product of the net torque 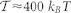 (thermal energy 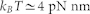) rotating the flagellar bundle and the body diameter *d* = 0.9 µm. However, this value of *q* is about two to three orders of magnitude larger than the fitted values in the far-field expression! Note that the above torque is close to the experimental value of 500 pN nm.^27^ The comparison reveals a discrepancy between our simulation results (and experimental estimates of the dipole strength) and the rotlet-dipole approach, which may arise from the overestimation of hydrodynamic interactions at short distances in the far-field approximation.

Equation (1) provides the characteristic slip length *b*_0_, which separates CW and CCW trajectories, i.e., yields a trajectory with vanishing average curvature. By setting *κ* = 0, we find 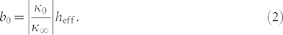
For the *E. coli* model with body length 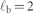, 3, and 4 µm at a distance of 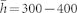 from the surface, *b*_0_ is about 40 nm and nearly independent of 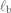, see Table 1. It is very interesting to see that a slip length of a few tens of nanometers can substantially alter the swimming behavior of *E. coli*. Therefore, we propose that *E. coli* can be employed as a natural sensor for the slip length of surfaces.

### Directed bacterial motion near patterned surfaces

**Figure 4:**
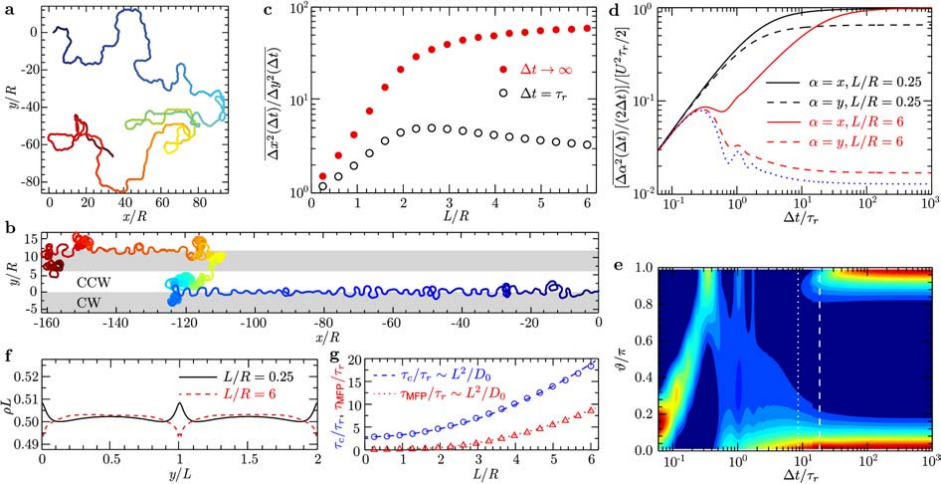
Active Brownian rod swimming on a ‘symmetric’ striped surface with curvature radii equal in magnitude but opposite in sign. Trajectories for (a) narrow (*L*/*R* = 0.25) and (b) broad (*L*/*R* = 6) stripes from simulations of duration *t* = 80*τ_r_* with starting points indicated in dark blue. The rotational diffusion time is *τ_r_* = 1/*D_r_* = 18 s (see Methods section). (c) The ratio of mean square displacement (MSD) parallel (*x*) and perpendicular (*y*) to the stripes as a function of stripe width *L* for Δ*t* = *τ_r_* and Δ*t* → ∞. (d) Rescaled MSD. The blue dotted line corresponds to a free circle swimmer.^34^ (e) Probability distribution of turning angle *ϑ* defined in Eq. (6) between successive trajectory segments of duration Δ*t* for *L*/*R* = 6. High and low probabilities are shown in red and blue, respectively. (f) Density profile *ρ* across two stripes. (g) Crossover time *τ_c_* between ballistic and diffusive regime along the stripes and the mean first-passage time *τ*_MFP_ for the rod to cross the stripe scale both quadratically with *L*. The white dashed and dotted lines in (e) indicate the corresponding *τ_c_* and *τ*_MFP_, respectively.

Motivated by the results for homogeneous surfaces, we propose that striped surfaces with different slip lengths can be used to direct bacterial motion. The alternating slip lengths of the stripes are chosen according to Eq. (1) such that *E. coli* swims in circles with opposite sign of curvature on neighboring stripes. This should result in an alternating trajectory along a stripe border, i.e., a 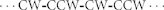 motion, see Fig. 4(b) and Movie S3 in *SI*. The dynamical behavior depends on the trajectory curvatures on the two types of stripes and the stripe width, as discussed in the following.

For the demonstration of the emergence of directed motion, we first consider stripes of equal width *L* and infinite length parallel to the *x*-axis, with curvatures |*κ*| = 1/*R* of equal magnitude. We model the bacterium as a self-propelled rod^33^,^34^ of length 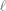 and swimming velocity *U* along its axis **e** = (cos *φ*, sin *φ*) with the orientation angle 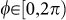. This allows us to reach the required large length- and time-scales in the simulations. For the symmetric setup of trajectory curvatures, the bacterium rotates at a rate Ω = Ω_0_ = *U*/*R* when it swims within CCW stripes and Ω = –Ω_0_ within CW stripes. When the bacterium crosses a CW-CCW border (e.g., at *y* = 0), we assume a linear profile Ω = Ω_0_*y*/*λ* with the width 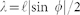 taking into account the orientation and finite size of the bacterium. The bacterium also exhibits translational and rotational diffusion with coefficients *D*_||_, 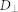, and *D_r_*. Further simulation details are described in the Methods section.

The swimming trajectories in Figs. 4(a) and 4(b) illustrate that the bacterial motion is rather isotropic for narrow stripes (*L*/*R* = 0.25), but becomes highly anisotropic along the *x*-axis for broad stripes (*L*/*R* = 6). In the latter case, we observe an oscillatory motion along stripe borders. To understand this behavior, we approximate the width *λ* by its average 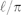. By neglecting noise terms in the equations of motion (5), we then obtain a pendulum equation 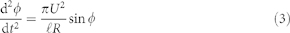
which implies a stable oscillation of the orientation angle *φ* around *φ* = *π* (i.e., the negative *x*-direction) at CW-CCW borders. Similarly, at CCW-CW borders (e.g., at *y* = *L*) oscillations around *φ* = 0 (i.e., the positive *x*-direction) are stable. The oscillatory motion is also seen with other choices of Ω(*y*), e.g., where 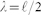, indicating that our results are robust with respect to modeling details. Figure 4(d) shows the mean square displacements (MSD) parallel and perpendicular to the stripes, 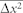 and 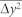, respectively. For narrow stripes, 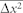 and 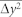 are almost equal and resemble the MSD of a persistent random walk, i.e., the MSD is ballistic for Δ*t* < *τ_r_* = 1/*D_r_* and diffusive for 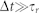. Independent of the stripe width, 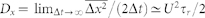. For broad stripes, however, 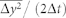 is orders of magnitude smaller than ±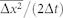, and *D_y_* converges with increasing *L*/*R* to the diffusion coefficient 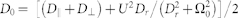 of a free circle swimmer.^34^ The transport on a substrate with broad stripes may be understood as follows: a bacterium diffuses with *D*_0_ within a stripe; once it hits the stripe border, it performs a directed snaking motion at the velocity *U* along *x*; the average duration of snaking is *τ_r_*, because there is no limit-cycle and snaking is neutrally stable; therefore, after *τ_r_* (on average) the bacterium starts to diffuse again within a stripe; as soon as the particle reach the neighboring border, motions along the positive and negative *x*-directions are equally probable and the transport along *x* gets diffusive. This becomes apparent from the distribution of the turning angles ϑ between successive displacements of duration Δ*t* (see Methods section), shown in Fig. 4(e) for *L*/*R* = 6, which characterizes the geometrical properties of a trajectory on various time scales. At short times Δ*t* < *τ_r_*, the characteristic angle with maximal probability drifts from *ϑ*_max_≈0 to *ϑ*_max_≈*π* due to free circular motion. On larger time scales, the motion gets first unidirectional (*ϑ*_max_≈0) and finally bidirectional (*ϑ*_max_≈0 and *ϑ*_max_≈*π* are equally probable). We calculate the crossover time *τ_c_* between the ballistic and diffusive regime along *x* as the time where the exponent *α* ≈ 1.4 in 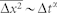. Moreover, we determine the mean first-passage time *τ*_M*FP*_, i.e., the mean time to cross the stripe. As can be seen from Fig. 4(g), both *τ_c_* and *τ*_M*FP*_ are proportional to *L*^2^/*D*_0_ and *τ_c_* > *τ*_M*FP*_, consistent with our conjecture; furthermore, *τ_c_* corresponds to the appearance of bidirectional motion, see the vertical white lines in Fig. 4(e). The absence of a limit-cycle at the stripe border becomes obvious from the nearly flat density distribution *ρ*(*y*) in Fig. 4(f). Finally, we show in Fig. 4(c) the ratio 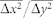 as a function of *L* at Δ*t* = *τ_r_* and Δ*t* → ∞. At the short time scale Δ*t* = *τ_r_*, the anisotropy assumes a maximum at 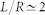. However, at long time scales, the diffusion anisotropy increases monotonically with *L*, exponentially for *L*/*R* ≤ 2 and then saturates for 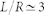.

**Figure 5:**
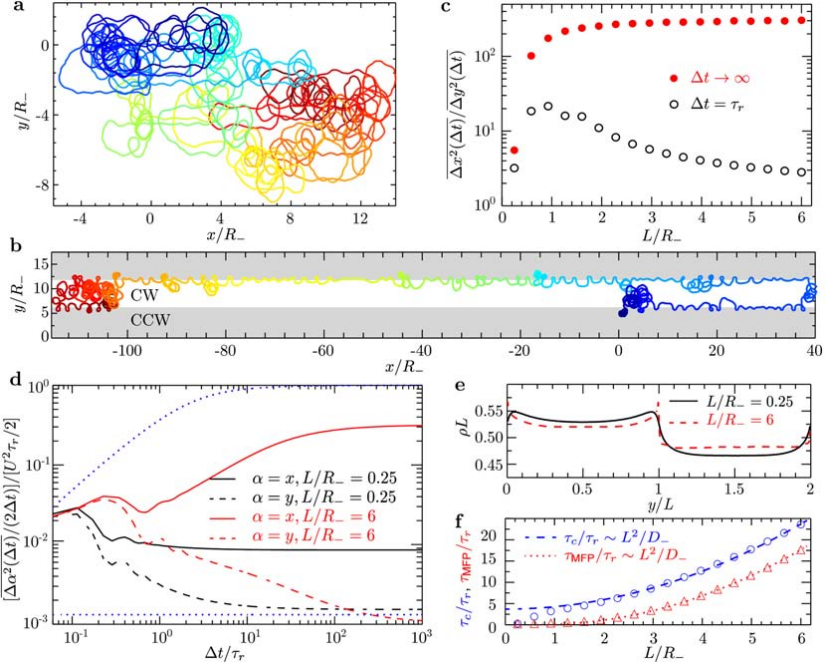
Active Brownian rod swimming on an ‘asymmetric’ striped surface with stripes of different curvatures (*R*_−_/*R*_+_ = 3). Trajectories for (a) narrow (*L*/*R*_−_ = 0.25) and (b) broad (*L*/*R*_−_ = 6) stripes from simulations of duration *t* = 80*τ_r_* with starting points indicated in dark blue. (c) Parallel-to-perpendicular MSD ratio as a function of stripe width *L* for Δ*t* = *τ_r_* and Δ*t* → ∞. (d) Rescaled MSD. The upper and lower blue dotted lines correspond to a persistent random walk and the diffusion coefficient *D*_−_*D*_+_/(*D*_−_ + *D*_+_), respectively. *D*_−_ and *D*_+_ are the diffusion coefficients of a free circle swimmer on the CW and CCW stripes. (e) Crossover time *τ_c_* between ballistic and diffusive regime along the stripes and the mean first-passage time *τ*_MFP_ as a function of *L*. (f) Density profile *ρ* across two stripes.

The setup of a symmetrically striped surface, with equal magnitude of the trajectory radius *R* on both stripes, is very special, because it would require a fine-tuning of slip lengths on the two kinds of stripes, and therefore a very particular selection of wall materials. Therefore, we study how asymmetrically striped surfaces modify the behavior. More precisely, we set *R*_−_ = 40 µm on CW stripes and *R*_+_ = *R_–_*/3 on CCW stripes. The trajectories in Figs. 5(a) and 5(b) show a similar behavior as in the case of a symmetric pattern, i.e., isotropic motion for narrow stripes and highly anisotropic along the *x*-axis for broad stripes. However, the anisotropy of the diffusion increases nearly five-fold, see Fig. 5(c). Furthermore, 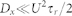 and *D_x_* decreases with decreasing *L*, see Fig. 5(d); *D_y_* converges to *D*_−_*D*_+_/(*D*_−_ + *D*_+_) with increasing *L* (*D*_−_ and *D*_+_ are the diffusion coefficients of a free circle swimmer in the CW and CCW stripe, respectively). The crossover time *τ_c_* ~ *L*^2^/*D*_−_ and mean first-passage time *τ*_M*FP*_ ~ *L*^2^/*D*_−_ are dominated by the faster diffusion process on the CW stripes with larger trajectory radius. In addition, the density distribution now displays different values in the two kinds of stripes because 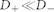, see Fig. 5(e). We conclude that the anisotropic transport over a striped surface is a very robust phenomenon.

## Discussion

We have presented a theoretical and numerical investigation of the effect of partial slip at surfaces on the curvature of swimming trajectories of flagellated bacteria. From a combination of mesoscale hydrodynamic simulations and scaling arguments, we predict that *E. coli* bacteria are able to sense surface slip on the nanoscale. An implication of our results is to use *E. coli* as a biosensor of surface slip. Moreover, an increasing slip length, which can be achieved experimentally by addition of grafted polymers, implies CCW circles of decreasing radius. Such tighter circles may enhance bacteria surface adsorption due to an increased local residence time; compare Figs. 1(b) and (d). Hence, physical sensing of high slip lengths may be paramount for biofilm formation and infection.

We further demonstrate that striped surfaces can be designed to direct bacterial motion along the stripe boundaries. The proposed striped surfaces with slip length smaller and larger than about 30 nm and width of order of 100 µm are accessible in laboratory experiments. Very importantly, the directed motion and anisotropic diffusion does not require a fine-tuning of slip lengths on the alternating stripe patterns, but is very robust and seems to only require different trajectory curvatures. Anisotropic transport even exists for stripes yielding the same sign of trajectory curvature (both CW or CCW) but different magnitudes. The resulting trajectory along the stripe border resembles a prolate cycloid. Therefore, transport along stripe boundaries is a very generic phenomenon.

First, it is important to emphasize that the anisotropic diffusion will persist at finite bacterial densities. In this case, a persistent multi-lane motion of bacteria can be predicted, in which individual bacteria occasionally change lanes, very similar as predicted above. Second, since the curvature radius of the swimming trajectory depends on the geometric parameters of the bacterium shape, this approach could also be employed for sorting bacteria according to size by an appropriate choice of stripe width. Bacteria with a smaller trajectory curvature would diffuse more isotropically, whereas bacteria with a larger trajectory curvature would diffuse more anisotropically along the stripe boundaries. Thus, after placing a mixture of both bacteria types on a small spot on the striped surface, bacteria with small and large trajectory curvatures will be found preferentially in a location in the *y*- and *x*-directions, respectively, at some distance from the initial location of the drop. In addition, a bacterial motion rectifier could be constructed from a transversally confined two-stripe system. In this case, there is only a single stripe boundary, so that all bacteria would move in the same direction in the center of the channel. Since there would also be directed motion along the wall,^13^ a more detailed study of such possible devices is required.

## Methods

### Mesoscale hydrodynamic simulations

The mesoscale hydrodynamic simulations combine coarse-grained molecular dynamics simulations for the bacterium and multiparticle collision dynamics method^14^,^15^ for the solvent. The model bacterium is constructed by coarse-grained particles, and consists of a spherocylindrical body and four helical flagellar filaments, see Fig. 1(a). The flagellar filament is based on the helical worm-like chain model.^24^,^25^ We choose the bacterial geometry and flagellar elastic properties as extracted from experiments of *E. coli* (*SI*). Each flagellum is driven by a motor torque 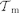 and the opposite torque is applied to the body to ensure that the bacterium is torque-free. We choose 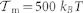, i.e., 2100 pN nm with Boltzmann constant *k_B_* and temperature *T* = 300 K, smaller than the stall torque of approximately 4500 pN nm^26^ of the flagellar motor. The flagellar bundle rotates at a frequency around 100 Hz and generates a propulsion force of about 0.6 pN, consistent with the experimental value of 0.57 pN.^27^

The surface is modeled by a hard wall with tunable boundary conditions. No-slip condition is implemented by applying bounce-back collision and virtual wall particles,^28^ whereas perfect-slip condition is obtained with specular reflection of fluid particles at the wall. The velocity of the fluid particle at the wall is inverted in bounce-back collision; in specular reflection, only the normal component of the velocity is inverted. A random mix of these two boundary conditions leads to partial slip.^29^ We systematically vary the mixing ratio to obtain slip lengths from 20 nm to 2 µm, a range readily accessible in experiments.^8^,^9^ We mimic the effect of additional surface-bacterium interactions such as van der Waals, electrostatic and steric interactions^30^,^31^ by a short-range repulsive potential 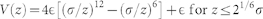 and *V*(*z*) = 0 otherwise, where 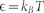 and the interaction range *σ* = 100 nm is set to avoid close contact of the bacterium with the surface. This short-range repulsion automatically takes care of possible bacterium-wall collision, which has been shown to be a possible cause of the accumulation of bacteria near walls.^20^,^32^,^33^ There is no attractive interaction potential between the bacterium and the surface. The simulations are done in cubic boxes of length 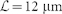 with periodic boundaries in *x*- and *y*-directions and two walls at *z* = 0 and 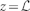.

### Analytical considerations

To derive an analytical expression for the curvature of the bacterial swimming trajectory, we consider a bacterium of length 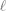 swimming with the velocity *U* parallel to the surface at a distance *h*, see Fig. 1(a). As illustrated in Fig. 3(b), the cell body of length 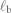 and diameter *d* rotates CCW around the *y*-axis at a rate *ω* and experiences two forces of hydrodynamic origin along the *x*-axis: (i) a pressure difference^21^ generated by the converging and diverging flows on either side of the gap between the body and the surface leads to a force 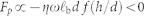, where *η* is the fluid viscosity, and the dimensionless function *f* has the properties *f*(*h*/*d*) → 0 for 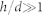 and *f*(*h*/*d*) → constant for 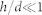;^23^ (ii) the fluid is sheared in the gap region, implying a velocity gradient and thus a shear force *F*_s_. By assuming a linear velocity profile *∂_z_u_x_* ≈ (_−_*ωd*/2)/(*h*_eff_ + *b*), where *h*_eff_ is the effective gap width and *b* the slip length of the surface, we obtain 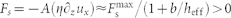, where 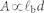 is the shear area and 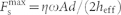 is the maximum shear force. These two forces add up to 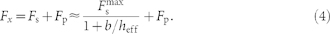
This expression describes the hydrodynamic interaction of rotating spherical, ellipsoidal, and spherocylindrical bodies with surfaces over a wide range of slip lengths very well, as shown by a comparison with existing numerical data in the range of 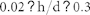 (Fig. S1 and Table S1 in *SI*).

Since the swimming bacterium is force-free, a force equal and opposite to Eq. (4) acts on the flagellar bundle, producing a torque 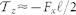 that drives the rotation of the cell around the *z*-axis at a rate 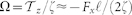, with the rotational friction coefficient 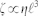. For a no-slip surface, Faxén's calculation^22^ implies that the force (4) translates the body in the direction of rolling along the surface, *i.e.* 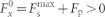, which is responsible for CW motion of the bacterium. For a perfect-slip surface, the force (4) is 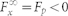, causing CCW motion. The curvature of a trajectory, *κ* = Ω/*U*, is then give by Eq. (1), which interpolates between 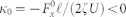 for no-slip surfaces (*b* = 0) and 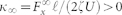 for perfect-slip surfaces (*b* = ∞). Here, *κ* < 0 denotes CW and *κ* > 0 CCW trajectories. We note that *κ* is independent of fluid viscosity *η* and swimming velocity *U*, since *ω* ∝ *U*.

### Brownian dynamics simulations

We consider a self-propelled rod swimming in two dimensions over a patterned surface and neglect the fluctuations of its distance from the surface, since the variation in the distance *h* of the bacterium to the surface from our hydrodynamic simulations is smaller than its length 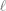, as shown in Fig. 2(e). The translational and rotational motions of the active rod are described according to 
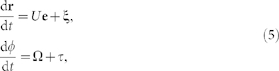
where **r** = (*x*, *y*) is the center of mass position, *U* is the swimming velocity along the orientation of the rod **e** = (cos *φ*, sin *φ*) with the orientation angle 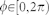. Here, ***ξ*** and *τ* are zero-mean Gaussian white noises of variance 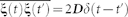 and 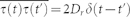. The diffusion tensor 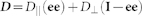 is given in terms of the parallel and perpendicular translational diffusion coefficients *D*_||_ and 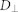. *D_r_* is the rotational diffusion coefficient. Using experimental results for *E. coli*,^2^,^20^,^27^ we set 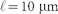, *U* = 20 µm/s, *R* = *U*/|Ω| = 40 µm, *D*_||_ = 0.14 µm^2^/s, 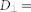 *D*_||_/2 and *D_r_* = 0.057 s^−1^.

### Geometrical characterization of trajectories

In order to characterize the geometrical properties of a swimming trajectory **r**(*t*) on various time scales, we examine the probability distribution function *P*(*ϑ*,Δ*t*) of turning angles ϑ between successive trajectory segments defined as

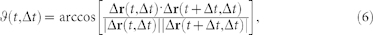
where Δ**r**(*t*, Δ*t*) = **r**(*t* + Δ*t*) − **r**(*t*) is the vector between two successive positions separated by the lag time Δ*t*;^35^ Δ*t* controls the degree of temporal coarse graining. The distribution function *P*(*ϑ*,Δ*t*) is flat for a homogenous diffusive motion due to the scale invariance of this process.

## Author Contributions

J.H., A.W., R.G.W. and G.G. designed the research, analyzed the data, and wrote the paper; J.H. and A.W. performed the research.

## Additional Information

**How to cite this article**: Hu, J., Wysocki, A., Winkler, R.G. & Gompper, G. Physical Sensing of Surface Properties by Microswimmers – Directing Bacterial Motion via Wall Slip. *Sci. Rep.* **5**, 9586; doi: 10.1038/srep09586 (2015).

## Supplementary Material

Supplementary Information Supplementary Information

Supplementary Information Supplementary Information

Supplementary Information Supplementary Information

Supplementary Information Supplementary Information

## Figures and Tables

**Table 1:** Properties of model *E. coli* swimming near a surface, as obtained from mesoscale hydrodynamic simulations. ℓ_b_: body length; ℓ/*d*: bacterial length to body diameter; *U*: swimming velocity; 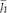: mean gap width; *h*_eff_: effective gap width; |*κ*_0_|^−1^ and |*κ*_∞_|^−1^: radius of curvature at *b* = 0 and *b* = ∞, respectively; *b*_0_: slip length of the surface, at which *E. coli* swims in a straight line. The ‘sim’ values for |*κ*_0_|^−1^ and |*κ*_∞_|^−1^ are directly measured from our simulations, ‘fit’ values are obtained from the fits in Fig. 3(a), and ‘exp’ are from experiments of *E. coli.*

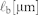	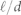	*U*[µm/s]	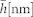	*h*_eff_[nm]	|*κ*_0_|^−1^[µm]			|*κ*_∞_|^−1^[µm]			
					Sim	Fit	exp^2^	Sim	Fit	exp^11^	*b*_0_[nm]
2	8.7	9.5 ± 0.8	307	182 ± 22	19 ± 1	21 ± 3		4.1 ± 0.1	4.2 ± 0.1		36 ± 3
3	9.8	9.1 ± 0.4	366	267 ± 23	33 ± 2	31 ± 3	≃10–50	4.2 ± 0.1	4.3 ± 0.1	≃5	38 ± 2
4	10.9	8.4 ± 0.2	390	370 ± 50	39 ± 3	40 ± 4		4.3 ± 0.1	4.3 ± 0.3		40 ± 3
