# Supplementary material for: Physical Sensing of Surface Properties by Microswimmers – Directing Bacterial Motion via Wall Slip
Source: Sci Rep. 2015 May 20;5:9586. doi: 10.1038/srep09586 (PMC4438609; doi:10.1038/srep09586)
Supplement: Supplementary Information [file srep09586-s1.pdf]

Supporting Information for “Physical Sensing of Surface Properties by Microswimmers – Directing Bacterial Motion via Wall Slip”

Jinglei Hu, Adam Wysocki, Roland G. Winkler & Gerhard Gompper*

Theoretical Soft Matter and Biophysics, Institute of Complex Systems and Institute for Advanced Simulation, Forschungszentrum Jülich, D-52425 Jülich, Germany

*Correspondence to g.gompper@fz-juelich.de.

Dependence of the hydrodynamic force on the surface slip length.—There are few studies devoted to the calculation of the hydrodynamic forces of a particle rotating parallel to a nearby partial-slip surface with prescribed slip length.[1,2] We compare Eq. (1) with existing numerical results for the force F_x on a non-translating spherical[1] or ellipsoidal[2] particle that rotates parallel to nearby surfaces with different slip lengths, see the notations in Fig. 3(b). Figure S1 shows that the numerical data points are well described by Eq. (1) with the fitted values of $F_{\rm s}^{\rm max}$, $F_{\rm p}$ and $h_{\rm eff}$ shown in Table S1. We find that the force for a no-slip surface $F_x^0 = F_{\rm s}^{\rm max} + F_{\rm p} > 0$ and the force for a perfect-slip surface $F_x^\infty = F_{\rm p} < 0$. The effective width $h_{\rm eff}$ from the linear approximation of the fluid velocity profile in the gap is comparable, but not equal to the gap width h. The comparison here confirms that Eq. (1) describes the hydrodynamic interaction of rotating spherical and ellipsoidal bodies with surfaces over a wide range of slip lengths very well. The slip length b_0, at which the force F_x vanishes, is also shown in Table S1. For a sphere of diameter $d = 0.9$ µm, one obtains $b_0 \approx 65$ nm at $h = 0.02265\,d = 20$ nm.

Mesoscale hydrodynamics simulations.—We briefly describe the hybrid simulation method here; for details, we refer to Refs. 3,4. The model *E. coli* has a spherocylindrical body and four left-handed helical filaments and is constructed by particles of mass M. We choose the body length $\ell_{\rm b} = 2-4$ µm, body diameter $d = 0.9$ µm, flagellar helix radius 0.2 µm, pitch 2.2 µm and angle 30° from experiments.[5,6] The elastic bending and twist moduli of the filaments are chosen according to the experimental range from about 10^{-24} to 10^{-21} N m^2.[7–9] The details of the model will be published elsewhere.

The solvent is modelled by a collection of point-like particles of mass m. Their dynamics comprises of alternating streaming and collision steps. In the streaming step, the solvent particles move ballistically and the position $\mathbf{r}_s$ of particle s with velocity $\mathbf{v}_s$ is updated according to $\mathbf{r}_s(t+\Delta t) = \mathbf{r}_s(t) + \mathbf{v}_s(t)\,\Delta t$ with Δt the time interval between collisions, while the dynamics of the body and flagellar particles is described by the Newton's equations of motion. In the collision step, all particles are sorted into cubic cells of length a and the velocity $\mathbf{v}_i$ of particle i in cell c is renewed via the collision rule[10,11]

$$\begin{aligned}\mathbf{v}_i^{\rm new} &= \mathbf{v}_c + \mathbf{v}_i^{\rm ran} - \sum_{j\in c} m_j \mathbf{v}_j^{\rm ran} \Big/ \sum_{j\in c} m_j \\ &+ \Big[I^{-1} \sum_{j\in c} m_j (\mathbf{r}_j - \mathbf{r}_c) \times (\mathbf{v}_j - \mathbf{v}_j^{\rm ran})\Big] \times (\mathbf{r}_i - \mathbf{r}_c),\end{aligned}$$

where $\mathbf{v}_c$ and $\mathbf{r}_c$ are the velocity and position of the center of mass of all particles in the cell c, m_j the mass of particle j in c, $\mathbf{v}_i^{\rm ran}$ a random velocity sampled from the Maxwell-Boltzmann distribution, and I the moment-of-inertia tensor of all particles in c. The collision rule (i) conserves both linear and angular momentum locally, *i.e.* in each collision cell, (ii) includes thermal fluctuations of the solvent, and (iii) maintains a constant temperature. To satisfy Galilean invariance, a random grid shift of the cells is performed before each collision step.[12]

Length and time scales.—By mapping the cell-body width $d = 9\,a$ to 0.9 µm for swimming *E. coli*[6], we obtain the length scale $a = 100$ nm, which sets the resolution of hydrodynamics in our simulation method. Comparison of the flagellar rotation rate $\omega_{\rm f} = 0.0356\sqrt{k_BT/ma^2}$ in our model to the experimental value of $2\pi \times 120$ Hz[6,13] leads to the time scale $\Delta t = 2.4$ µs.

Simulation setup.—Our simulations are performed in cubic boxes of length $\mathcal{L} = 120\,a$ with periodic boundaries in the x- and y-directions and two planar surfaces implemented at $z = 0$ and $z = \mathcal{L}$. Additional simulations with $\mathcal{L} = 150\,a$ are run and the obtained average curvature is consistent with that from $\mathcal{L} = 120\,a$ within numerical errors, indicating the periodic boundaries do not affect our simulation results. We choose the collision time step $\Delta t = 0.05\sqrt{ma^2/k_BT}$ and the solvent density $\rho = 10\,m/a^3$, leading to the solvent viscosity $\eta = 7.15\sqrt{mk_BT}/a^2$ and the Schmidt number $Sc = 20$, for which momentum transport dominates over mass transport. Newton's equations of motion for the particles of the *E. coli* model are integrated with a time step $\delta t = \Delta t/25$ using the velocity-Verlet algorithm.

Slip length of the surface.—We obtain partial-slip surfaces with different slip lengths by randomly mixing no-slip and

perfect-slip boundary conditions. Figure S2 shows the slip length b as a function of the mixing ratio p, defined as the probability of applying no-slip boundary condition at each collision step ($1-p$ for perfect-slip). b is measured from the velocity gradient of the fluid under shear.

References

1. Luo, H., Pozrikidis, C. Effect of surface slip on stokes flow past a spherical particle in infinite fluid and near a plane wall. *J. Eng. Math.* **62**,1-21 (2008).
2. Ghalya, N., Sellier, A., Feuillebois, F. Migration of a solid and arbitrarily-shaped particle near a plane slipping wall. *J. Phys.: Conf. Ser.* **392**, 012013 (2012).
3. Kapral, R. Multiparticle collision dynamics: Simulation of complex systems on mesoscales. *Adv. Chem. Phys.* **140**, 89-146 (2008).
4. Gompper, G., Ihle, T., Kroll, D. M., Winkler, R. G. Multi-particle collision dynamics: A particle-based mesoscale simulation approach to the hydrodynamics of complex fluids. *Adv. Polym. Sci.* **221**, 1-87 (2009).
5. Turner, L., Ryu, W. S., Berg, H. C. Real-time imaging of fluorescent flagellar filaments. *J. Bacteriol.* **182** 2793-2801 (2000).
6. Darnton, N. C., Turner, L., Rojevsky, S., Berg, H. C. On torque and tumbling in swimming escherichia coli. *J. Bacteriol.* **189**, 1756-1764 (2007).
7. Fujime, S., Maruyama, M., Asakura, S. Flexural rigidity of bacterial flagella studied by quasielastic scattering of laser light. *J. Mol. Biol.* **68**, 347-359 (1972).
8. Trachtenberg, S., Hammel, I. The rigidity of bacterial flagellar filaments and its relation to filament polymorphism. *J. Struct. Biol.* **109**, 18-27 (1992).
9. Darnton, N.C., Berg, H.C. Force-extension measurements on bacterial flagella: Triggering polymorphic transformations. *Biophys. J.* **92**, 2230-2236 (2007).
10. Götze, I. O., Noguchi, H., Gompper, G. Relevance of angular-momentum conservation in mesoscale hydrodynamics simulations. *Phys. Rev. E* **76**, 046705 (2007).
11. Noguchi, H., Gompper, G. Transport coefficients of off-lattice mesoscale-hydrodynamics simulation techniques. *Phys. Rev. E* **78**, 016706-12 (2008).
12. Ihle, T., Kroll, D. M. Stochastic rotation dynamics: A galilean-invariant mesoscopic model for fluid flow. *Phys. Rev. E* **63**, 020201-4 (2001).
13. Chattopadhyay, S., Moldovan, R., Yeung, C., Wu, X. L. Swimming efficiency of bacterium *Escherichia coli. Proc. Natl. Acad. Sci. USA* **103**, 13712-13717 (2006).

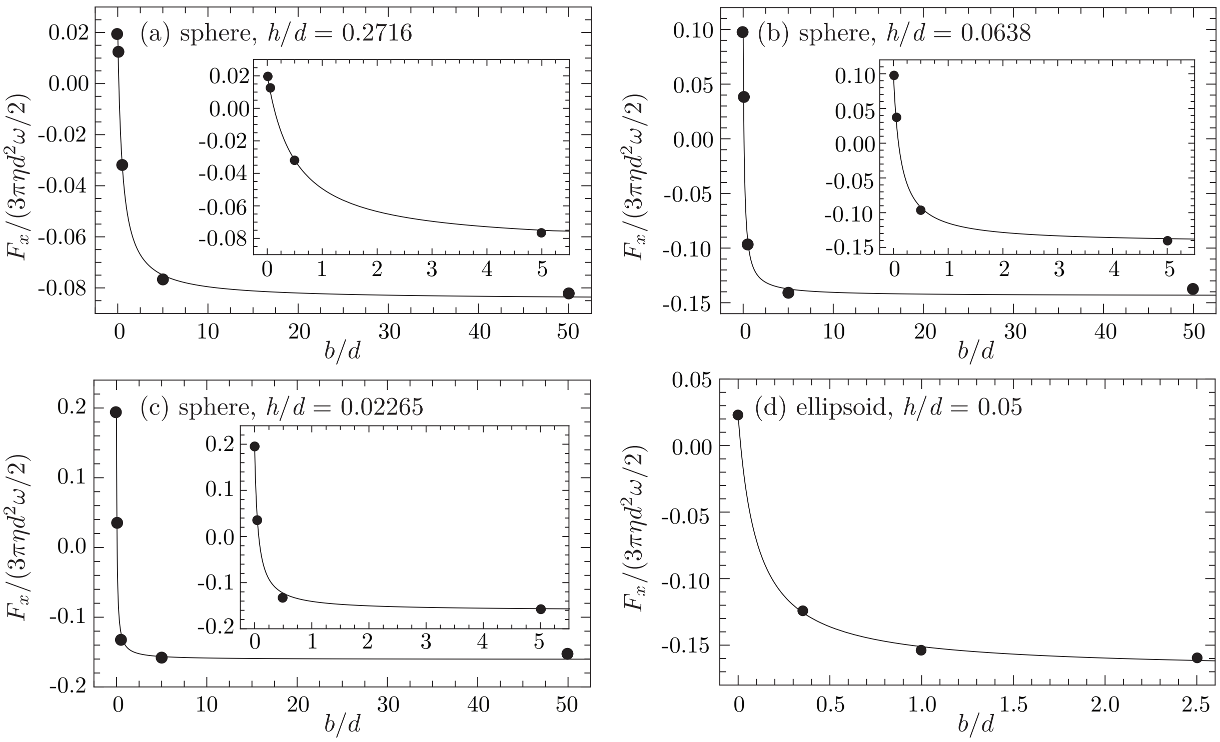

Figure S1: Force F_x *vs.* slip length b for (a)-(c) a sphere of diameter d (from Ref. 1) and (d) an ellipsoid with axes $a_x = d$, $a_y = 6d/5$ and $a_z = 5d/6$ (from Ref. 2) rotating parallel to a surface at a distance h. The solid lines are least-square fits of data points to Eq. (2) with the fitted values shown in Table S1. Insets in (a)-(c) are close-up of the first four points. See Fig. 3(b) for the notations.

	h/d	h_{eff}/h	$F_s^{\max}$	F_p	b_0/h
	0.2716	1.85	0.105	-0.085	0.45
Sphere	0.0638	2.09	0.244	-0.144	1.46
	0.02265	2.62	0.356	-0.161	3.18
Ellipsoid	0.05	2.08	0.192	-0.169	0.28

Table S1: Properties of a particle rotating parallel to a nearby surface, as obtained from the fits in Fig. S1. $F_s^{\max}$ and F_p are rescaled by $3\pi\eta d^2\omega/2$.

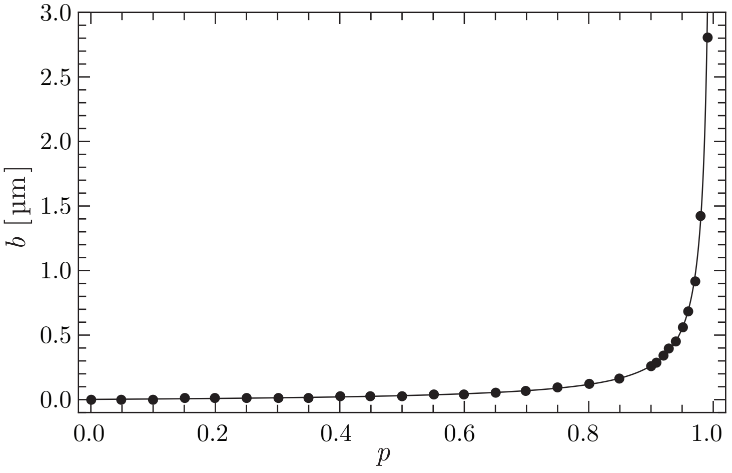

Figure S2: Slip length b of the surface *vs.* probability p of applying no-slip boundary condition ($1-p$ for perfect-slip) at each collision step in our MPC simulations. The solid line is a guide to the eye.

Movie S1: Simulation animation of model *E. coli* swimming in a bulk fluid. The swimming motion is preceded by bundling of initially separated flagella. Viewed from the front, the flagellar bundle rotates clockwise driven by the motor torque, and the body counter-rotates to balance the torque on the flagella. The sphero-cylindrical cell body has a length of 2 µm and a diameter of 0.9 µm. Each flagellar filament is a three-turn left-handed helix of radius 0.2 µm, pitch 2.2 µm and angle 30°. The length of the bacterium is about 8 µm. For clarity, the fluid is not shown here.

Movie S2: Simulation animation of *E. coli* swimming near a no-slip surface. The circular trajectory with clockwise motion is viewed from above the surface, compare also Fig. 2(a). The geometry of the bacterium is the same as in Fig. 1(a) and Movie S1.

Movie S3: Simulation animation of active Brownian rod swimming near a striped surface. The trailing path in color (red to blue) represents the swimming trajectory for the past 40 seconds. R_- and R_+ are the radii of curvature for the clockwise and counterclockwise trajectories on the alternating stripes with width L, respectively. The geometry, swimming velocity and diffusion coefficients of the active rod are in agreement with experimental values of *E. coli*.
